# Association between weight change and risk of metabolic abnormalities in non-overweight/obese and overweight/obese population: A retrospective cohort study among Chinese adults

**DOI:** 10.3389/fendo.2022.1029941

**Published:** 2022-12-20

**Authors:** Yanyan Dai, Yujuan Li, Shu Yang, Weiwei Xu, Hong Jia, Chao Yang

**Affiliations:** ^1^ School of Public Health, Southwest Medical University, Luzhou, Sichuan, China; ^2^ Health Management Center, The Affiliated Hospital of Southwest Medical University, Luzhou, China; ^3^ School of Intelligent Medicine, Chengdu University of Traditional Chinese Medicine, Chengdu, Sichuan, China

**Keywords:** metabolic abnormalities, weight gain, weight loss, non-overweight/obesity, overweight/obesity

## Abstract

**Objectives:**

To explore the effects of weight change on the risk of metabolic abnormalities in the Chinese population.

**Methods:**

A total of 1895 metabolically healthy adults aged 21–78 years completed anthropometric and biological measurements at baseline (2012) and at an eight year follow-up (2020). Based on absolute weight change and relative weight change, the participants were split into five classes. A Cox proportional hazards regression model was used to estimate the relative risk (RR) and 95% confidence intervals (95% CI) for the risk of metabolic abnormalities using stable weight as the reference group. Stratified analysis was used to explore this relationship in participants with different baseline body mass index (BMI) levels.

**Results:**

During the follow-up period, 35.41% of the participants retained a stable weight, and 10.71% had metabolic abnormalities. After covariate adjustment, for every kilogram gained over eight years, the risk of developing metabolic abnormalities increased by 22% (RR: 1.094; 95% CI: 1.063–1.127). Compared with stable weight participants, weight gain of 2–4 Kg and weight gain ≥ 4 Kg exhibited significantly higher risks of metabolic abnormalities, with RR of 1.700 (95% CI 1.150–2.513) and 1.981 (95% CI 1.372–2.859), respectively. A weight gain of ≥ 4 Kg had an opposite effect on the overweight/obesity and non-overweight/obesity groups, with an increased risk of metabolic abnormalities only in the non-overweight/obesity group (RR, 2.291; 95% CI, 1.331–3.942). Moreover, weight loss ≥ 4 Kg significantly reduced the risk of metabolic abnormalities only among overweight/obese adults (RR 0.373; 95% CI 0.154–0.906). Similar results were observed in relative body weight change analyses.

**Conclusions:**

Long-term excessive body weight gain is positively associated with an increased risk of metabolic abnormalities among adults with non-overweight/obesity, whereas long-term body weight loss is a protective factor for metabolic health among adults with overweight/obesity.

## 1 Introduction

Metabolic syndrome (MetS) is a complex metabolic abnormality that includes hyperglycemia, elevated blood pressure, elevated triglyceride levels, low high-density lipoprotein cholesterol (HDL-C), and obesity (1–3). It is associated with 76% of cardiovascular disease cases (4), 47% all-cause mortality (5), and 33% elevated cancer mortality (6). Therefore, it has become a growing public health problem worldwide (7).

Numerous studies on Western adults have indicated that high body weight and excessive body weight gain may increase the risk of MetS (8–14), whereas weight loss reduces this risk (15–20). However, few cohort studies have focused on this association in Chinese adults (21, 22). Yuan et al. found that the risk of MetS increased with weight gain and decreased with weight loss in Chinese adults (21), and Lin et al. observed an association between weight gain and the risk of MetS in Taiwanese individuals (22). However, the follow-up periods in both studies were relatively short, which may have reduced the robustness of the findings. After stratifying the population by overweight status, current studies have shown inconsistent results regarding the relationship between weight change and the risk of metabolic abnormalities (11, 23–25). For the overweight/obese population, previous studies showed that the results ranged from the inverted U-shaped association of weight gain with MetS (23) to the less association (11). Moreover, few Chinese cohort studies have explored this association separately in populations with different body mass indices (BMI).

Therefore, we used the data from 2012 to 2020 in a retrospective cohort study to explore the association between weight change and the risk of metabolic abnormalities in Chinese adults. Additionally, we sought to identify whether weight change produces different risks for metabolic abnormalities in different BMI strata.

## 2 Methods

### 2.1 Study population

**Figure 1:**
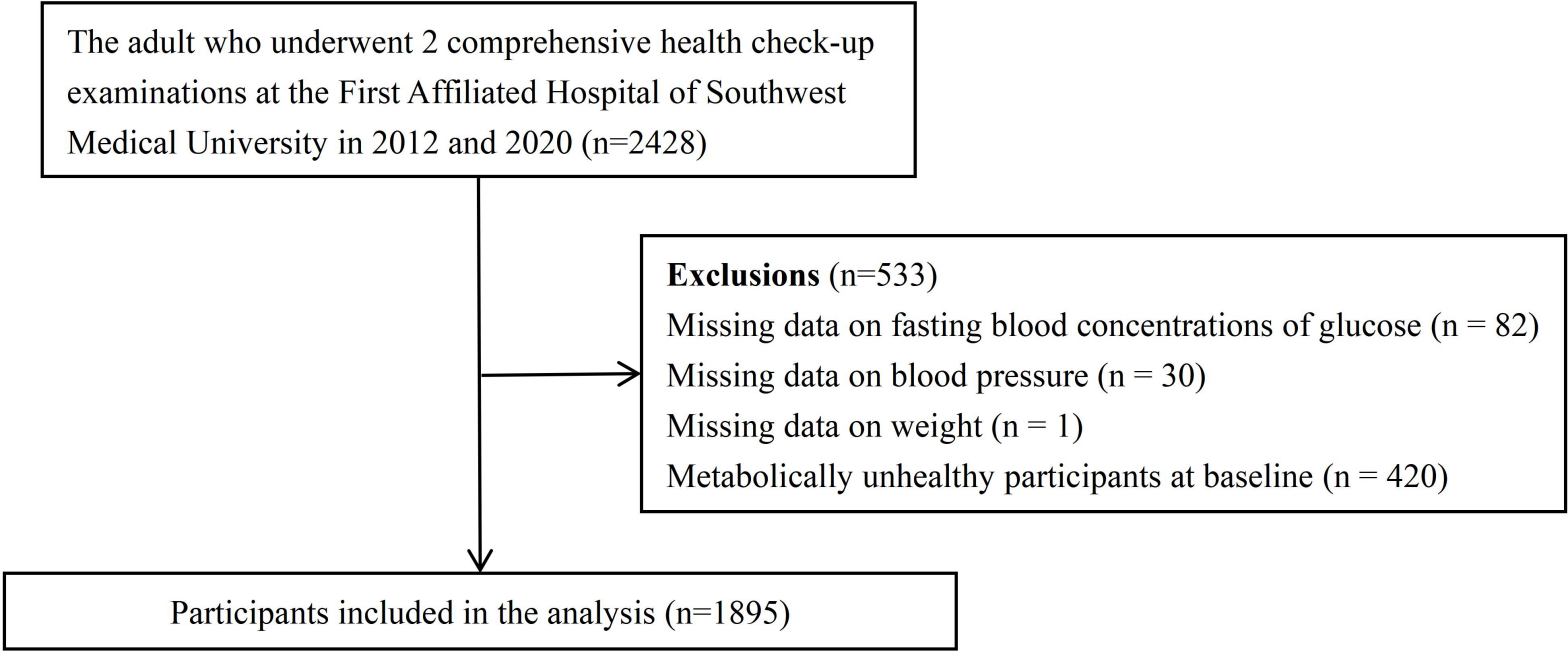
Flow chart of study participants. (n=1895).

The enrolment process for this cohort is shown in **Figure 1**. We initially considered 2428 individuals aged 21–78 years. Our study was based on the results of health examinations conducted in 2012 and 2020 at the First Affiliated Hospital of Southwest Medical University, Luzhou, China, which screened all employees from seven companies. Employees of these companies have a free diet and do not follow a particular dietary regimen. In total, 533 individuals were excluded for the following reasons:82 lacked fasting blood glucose concentrations; 30 lacked systolic blood pressure or diastolic blood pressure measurements; 1 lacked weight measurement; and 420 were diagnosed with metabolic abnormalities based on fasting blood concentrations of glucose, triglycerides, high-density lipoprotein (HDL), systolic blood pressure, and diastolic blood pressure in 2012. Consequently, 1895 subjects (1018 males and 877 females) with baseline metabolic health were enrolled. Before agreeing to the physical examination, each subject provided verbal consent after being fully informed. The First Affiliated Hospital’s Clinical Medicine College Ethics Committee at Southwest Medical University approved this study.

### 2.2 Anthropometric and biological

Anthropometric measurements included height and body weight. The participants were required to wear light clothing, no shoes, and no hats. Weight and height were accurate to the nearest 0.1 kg and 0.1 cm, respectively.

Participants were instructed to abstain from smoking and consuming alcohol, coffee, and tea before the blood pressure measurement. During the measurement, the subjects sat comfortably with their arm positioned at the level of the heart. According to the American Heart Association’s standardized protocol, blood pressure was measured using an electronic oscillometric blood pressure measurement device (HEM-770 AFuzzy, Omron, Kyoto, Japan) by trained health professionals. The measurements were repeated three times with a 30 s interval between each measurement, and the average was used for analysis.

The participants underwent biochemical tests with unified devices, using whole blood samples. The test was given between 8:00 and 11:00 a.m. Participants were instructed to fast for at least 12 h prior to the test and to refrain from physical activity the day before. Biochemical parameters included total cholesterol (TC), triglycerides (TG), high-density lipoprotein cholesterol (HDL-c), fasting plasma glucose (FPG), creatinine (Cr), blood urea nitrogen (BUN), serum uric acid (SUA), aspartate aminotransferase (AST), and alanine aminotransferase (ALT). An automatic biochemical analyzer was used to measure all biochemical parameters (SIEMENS ADVIA2400).

### 2.3 Definition of BMI and weight change

BMI was calculated using height and weight (kg/m2). We defined overweight/obesity as a BMI ≥24 kg/m2 based on the China Obesity Task Force recommendations for the Chinese adult population (26).

Based on previous studies (27), absolute body weight change was calculated using the following equations: body weight in 2020 minus body weight in 2012. We divided the eight-year weight change into five clinically meaningful groups: loss or gain < 2 Kg (Stable), loss 2–4 Kg (Mild loss), loss ≥ 4 Kg (Medium or large loss), gain 2–4 Kg (Mild gain) and gain ≥ 4 Kg (Medium or large gain). Relative body weight change was calculated using the following equation: (body weight in 2020 minus body weight in 2012)/body weight in 2012. Participants were classified into five groups for further analyses based on relative body weight change: loss or gain of 3% (Stable), loss of 3 to 6% (Mild loss), loss of ≥ 6% (Medium or large loss), gain of 3 to 6% (Mild gain), and gain ≥ 6% (Medium or large gain).

### 2.4 Definition of metabolic abnormalities

The change in body weight is related to BMI, and our study was stratified according to BMI. Therefore, we utilized the diagnostic criteria of the Chinese Diabetes Society (CDS) and defined metabolic abnormalities as the presence of ≥2 of the following 4 indicators: (i) fasting plasma glucose (FPG) ≥ 6.1 mmol/L (110 mg/dL) or 2h PG ≥ 7.8 mmol/L (140 mg/dL) or previously diagnosed and treated Type 2 diabetes; (ii) blood pressure ≥ 140/90 mmHg or on antihypertensive drug treatment in a patient with a history of hypertension; (iii) triglycerides ≥ 1.70 mmol/L (150 mg/dL); or (iv) HDL cholesterol < 1.00 mmol/L (39 mg/dL) in women, or < 0.90 mmol/L (35 mg/dL) in men (28).

### 2.5 Statistical methods

Quantitative variables for the baseline characteristics of the study population are presented as median and interquartile range or mean ± standard deviation, and qualitative variables are expressed as percentages. According to the normality of the data, quantitative variables were compared using one-way ANOVA or the Kruskal-Wallis test. Qualitative variables were compared using the chi-squared test. We assessed the association between body weight change and incidence of metabolic abnormalities at follow-up using the Cox proportional hazards regression model. A Cox proportional hazards regression model was used to estimate the relative risk (RR) and 95% confidence intervals (CIs) for the risk of metabolic abnormalities by groups of absolute body weight change and relative body weight change, with loss or gain of 2 Kg or 3% as the reference. The results of the relative weight change analysis were used as a sensitivity analysis to test the robustness of our findings.

All covariates were selected based on the known risk factors for MetS. We used four models with increasing degrees of adjustment to account for potential confounders at the baseline. We then stratified the participants by BMI status at baseline for subgroup analysis.

All statistical analyses were performed using the SPSS Package version 26.0. Statistical significance was set at P < 0.05 (two-tailed).

## 3 Results

At baseline, the average age was 40.63 years (SD 11.04) among the 1895 participants (1018 men and 877 women) with an average BMI of 22.34 kg (SD 2.91). Based on recommended BMI cutoffs by the Working Group on Obesity in China, the proportion of non-overweight/obese and overweight/obese adults was 72.72% and 27.28%, respectively. The baseline characteristics according to the weight change category are shown in **Table 1**. More than one-third of the participants (35.41%) retained a stable body weight, 28.34% had excessive weight gain ≥ 4 Kg, and only 7.07% had lost 4 Kg or less of their baseline body weight. The differences in sex ratio, average age, TC, FPG, TG, HDL-C, LDL-C, BUN, Cr, UA, ALT, AST, SBP, and BMI were significant between the five groups, but no significant difference was observed in diastolic blood pressure (DBP).

**Table 1:** Baseline characteristics of study participants by body weight change.

Baseline characteristics	All subjects (n = 1895)	Stableloss or gain < 2 Kg (n = 671)	Mild lossloss 2–4 Kg (n = 186)	Medium or largeloss ≥ 4 Kg (n = 134)	Mild gaingain 2–4 Kg (n = 367)	Medium or largegain ≥ 4 Kg (n = 537)	pvalue
Female (%)	877 (46.28)	323 (48.14)	66 (35.48)	56 (41.79)	181 (49.32)	251 (46.74)	0.016
Age (years)	40.63 ± 11.04	42.66 ± 10.67	45.87 ± 11.50	44.17 ± 12.00	39.10 ± 10.28	36.46 ± 9.90	<0.001
TC (mmol/L)	4.68 ± 0.82	4.71 ± 0.80	4.89 ± 0.85	4.87 ± 0.90	4.64 ± 0.77	4.55 ± 0.80	<0.001
FPG (mmol/L)	4.95 ± 0.65	4.98 ± 0.63	5.05 ± 0.68	5.12 ± 0.81	4.92 ± 0.71	4.85 ± 0.56	<0.001
TG (mmol/L)	1.19 (0.88-1.59)	1.17 (0.87-1.58)	1.30 (1.00-1.82)	1.40 (1.08-1.98)	1.20 (0.90-1.61)	1.12 (0.82-1.48)	<0.001
HDL-C (mmol/L)	1.47 ± 0.32	1.48 ± 0.32	1.43 ± 0.29	1.39 ± 0.29	1.48 ± 0.32	1.48 ± 0.31	0.009
LDL-C (mmol/L)	2.49 ± 0.66	2.49 ± 0.64	2.66 ± 0.67	2.66 ± 0.72	2.44 ± 0.62	2.41 ± 0.67	<0.001
BUN (mmol/L)	5.06 ± 1.27	5.07 ± 1.30	5.36 ± 1.40	5.09 ± 1.32	4.90 ± 1.20	5.03 ± 1.22	0.002
Cr (μmol/L)	68.60 (57.40-80.10)	68.20 (56.80-80.10)	73.05 (58.38-83.65)	71.50 (58.65-82.75)	68.20 (56.30-79.20)	68.00 (57.95-79.35)	0.047
UA (μmol/L)	328.28 ± 84.45	324.44 ± 84.30	343.18 ± 84.23	349.49 ± 89.00	323.83 ± 83.82	325.67 ± 82.91	0.002
ALT (g/L)	20.20 (14.80-29.90)	19.50 (14.40-29.20)	22.75 (17.08-34.90)	22.50 (15.20-32.25)	19.70 (15.00-28.80)	19.90 (14.35-29.40)	0.003
AST (g/L)	23.50 (20.20-28.40)	23.50 (20.40-28.70)	24.85 (21.28-30.08)	24.50 (20.68-28.75)	22.80 (19.60-27.10)	23.30 (20.15-28.20)	0.004
SBP (mmHg)	115.59 ± 15.84	116.33 ± 16.00	118.24 ± 16.84	118.01 ± 16.01	114.37 ± 15.88	113.97 ± 15.01	0.001
DBP (mmHg)	77.77 ± 9.89	78.07 ± 9.52	79.23 ± 9.90	79.06 ± 10.94	76.96 ± 10.36	77.13 ± 9.66	0.210
BMI (kg/m^2^)	22.34 ± 2.91	22.37 ± 2.91	23.16 ± 2.60	24.36 ± 3.30	21.84 ± 2.68	21.87 ± 2.77	<0.001

### 3.1 Association of weight change with metabolic results over eight years

**Figure 2:**
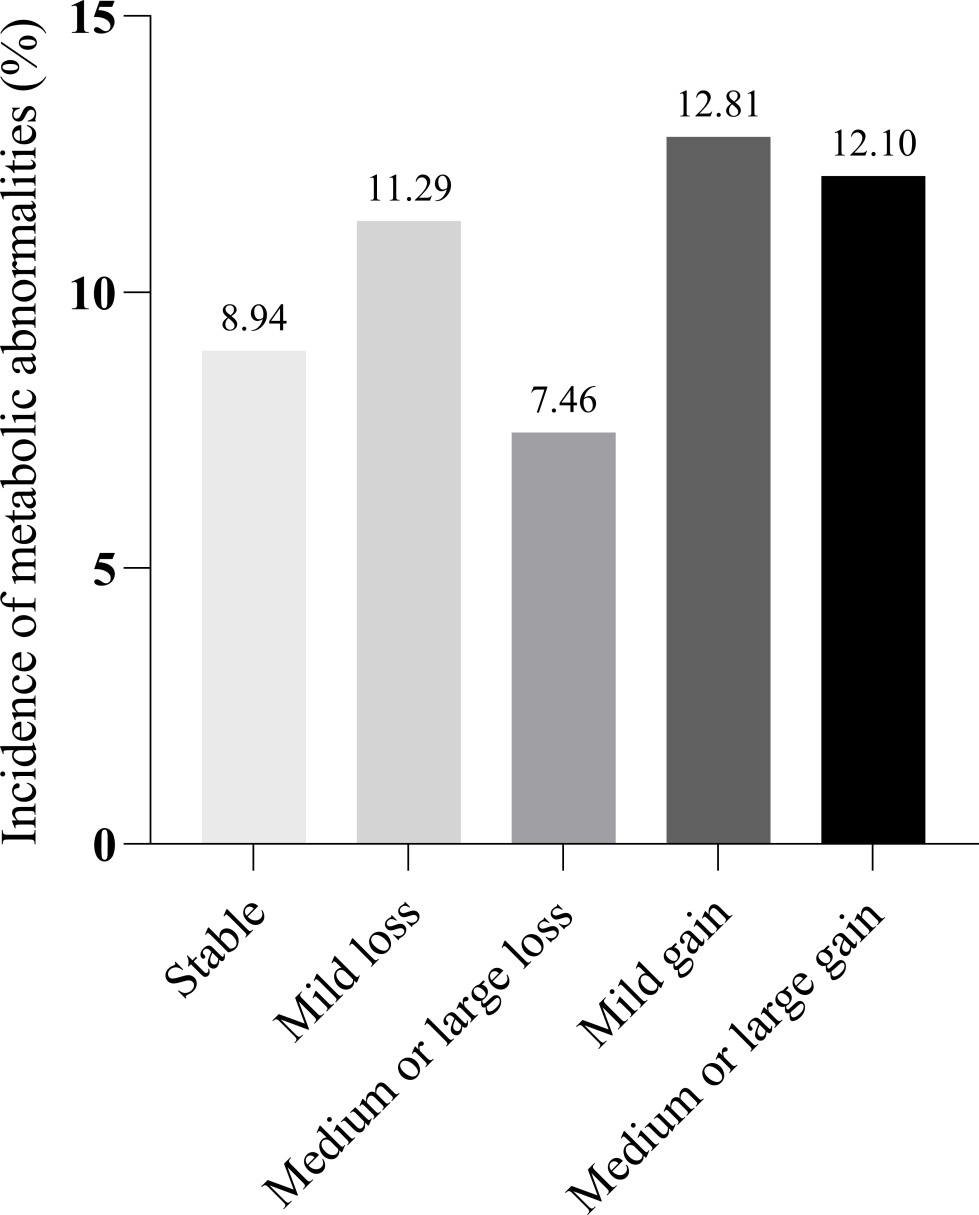
Incidence of metabolic abnormalities by weight change group. Stable: Weight loss or gain <2 kg. Mild loss: Weight loss 2 to 4 kg. Medium or large loss: Weight loss ≥4kg. Mild gain: Weight gain 2 to 4kg. Medium or large gain: Weight gain ≥4kg.

In total, 10.71% (203 of 1895) of the participants developed metabolic abnormalities during the eight-year follow-up. In addition, 8.94% of the participants with weight stability had metabolic abnormalities. The incidence of participants with metabolic abnormalities in the other groups (Mild loss, Medium or large loss, Mild gain, and Medium or large gain) was approximately 10% (11.29%, 7.46%, 12.81%, and 12.10%, respectively) (**Table 2** and **Figure 2**). Absolute body weight change was positively associated with the incidence of metabolic abnormalities in both univariate and multivariate models. A 1 Kg increase in body weight during follow-up predicted 9.40% of metabolic abnormalities. We further classified weight changes to assess the relationship between dynamic absolute weight change, dynamic relative weight change, and the occurrence of metabolic abnormalities. Compared with the Stable group, Mild gain and Medium or large gain had increased metabolic abnormalities (adjusted RR 1.700 [95% CI 1.150-2.513] and 1.981 [95% CI 1.372-2.859], Model 4). Similar results were obtained for relative body weight change (**Tables 2** and **S1**).

**Table 2:** Associations between absolute body weight change and risk of metabolic abnormalities.

Variables	No. of cases (%)	Model 1	Model 2	Model 3	Model 4
Absolute body weight change^a^	203 (10.71)	1.037 (1.009-1.065)	1.066 (1.035-1.097)	1.084 (1.053-1.115)	1.094 (1.063-1.127)
Stable	60 (8.94)	1	1	1	1
Mild loss	21 (11.29)	1.263 (0.768-2.075)	1.073 (0.652-1.768)	1.166 (0.705-1.926)	1.058 (0.638-1.754)
Medium or large loss	10 (7.46)	0.835 (0.427-1.630)	0.760 (0.389-1.485)	0.579 (0.295-1.135)	0.519 (0.263-1.022)
Mild gain	47 (12.81)	1.432 (0.978-2.098)	1.631 (1.111-2.395)	1.579 (1.073-2.323)	1.700 (1.150-2.513)
Medium or large gain	65 (12.10)	1.354 (0.953-1.923)	1.687 (1.177-2.416)	1.898 (1.319-2.731)	1.981 (1.372-2.859)

Stable: Weight loss or gain <2 kg. Mild loss: Weight loss 2 to 4 kg. Medium or large loss: Weight loss ≥4kg. Mild gain: Weight gain 2 to 4kg. Medium or large gain: Weight gain ≥4kg.

^a^Absolute body weight change was calculated by the following equations: body weight (2020) - body weight (2012).

Data are RR and 95% confidence intervals (CIs).

Model 1 has been body weight adjusted.

Model 2: Factors from Model 1 as well as baseline gender and age were adjusted.

Model 3: Factors from Model 2 as well as FPG, TG, HDL-c, and SBP at baseline were adjusted.

Model 4: Factors from Model 3 as well as baseline values of TC, LDL-c, BUN, Cr, SUA, ALT, AST, and BMI were adjusted.

### 3.2 Association of change in different BMI strata with risk of metabolic abnormalities

The incidence of metabolic abnormalities was 7.18% in participants with BMI < 24 at baseline. Absolute body weight change was positively associated with the incidence of metabolic abnormalities in both univariate and multivariate models. Compared with the Stable group, Medium or large gain significantly increased the risk of metabolic abnormalities (adjusted RR 2.291 [95% CI 1.331-3.942], Model 4) for people with BMI < 24 at baseline. However, the risk of metabolic abnormalities was not significantly reduced for those who changed to the weight loss group compared to weight gain and stable body weight groups in both univariate and multivariate models. Similar results were found in the relative body weight change analysis (**Tables 3** and **S2**).

**Table 3:** Associations between absolute body weight change and risk of metabolic abnormalities in subjects with non-overweight/obesity.

Variables	No. of cases (%)	Model 1	Model 2	Model 3	Model 4
Absolute body weight change^a^	99 (7.18)	1.056 (1.016-1.097)	1.084 (1.043-1.127)	1.089 (1.047-1.131)	1.092 (1.048-1.137)
Stable	24 (4.92)	1	1	1	1
Mild loss	9 (7.89)	1.605 (0.746-3.453)	1.353 (0.625-2.927)	1.664 (0.754-3.675)	1.409 (0.626-3.170)
Medium or large loss	4 (5.97)	1.214 (0.421-3.499)	1.004 (0.346-2.908)	1.106 (0.375-3260)	0.860 (0.290-2.547)
Mild gain	25 (8.80)	1.790 (1.022-3.134)	2.063 (1.174-3.623)	1.924 (1.090-3.396)	1.775 (0.995-3.165)
Medium or large gain	37 (8.70)	1.770 (1.059-2.959)	2.177 (1.290-3.673)	2.515 (1.463-4.321)	2.291 (1.331-3.942)

Stable: Weight loss or gain <2 kg. Mild loss: Weight loss 2 to 4 kg. Medium or large loss: Weight loss ≥4kg. Mild gain: Weight gain 2 to 4kg. Medium or large gain: Weight gain ≥4kg.

^a^Absolute body weight change was calculated by the following equations: body weight (2020) - body weight (2012).

Data are RR and 95% confidence intervals (CIs).

Model 1 has been body weight adjusted.

Model 2: Factors from Model 1 as well as baseline gender and age were adjusted.

Model 3: Factors from Model 2 as well as FPG, TG, HDL-c, and SBP at baseline were adjusted.

Model 4: Factors from Model 3 as well as baseline values of TC, LDL-c, BUN, Cr, SUA, ALT, and AST,were adjusted.

Among participants with BMI ≥ 24 at baseline, the incidence of metabolic abnormalities (20.12%) was higher than that in subjects without overweight/obesity. Absolute body weight change was positively associated with the incidence of metabolic abnormalities in univariate and multivariate models. Compared with the Stable group, Medium or large weight loss reduced the risk of metabolic abnormalities (adjusted RR 0.373 [95% CI 0.154-0.906], Model 4) for people with overweight/obesity at baseline. However, the risk of metabolic abnormalities was not significantly increased in the weight gain group compared with the non-weight gain and stable body weight groups in univariate and multivariate models. Similar results were found in the relative body weight change analysis (**Tables 4** and **S3**).

**Table 4:** Associations between absolute body weight change and risk of metabolic abnormalities in subjects with overweight/obesity.

Variables	No. of cases (%)	Model 1	Model 2	Model 3	Model 4
Absolute body weight change^a^	104 (20.12)	1.054 (1.014-1.096)	1.075 (1.029-1.124)	1.096 (1.047-1.147)	1.106 (1.054-1.160)
Stable	36 (19.67)	1	1	1	1
Mild loss	12 (16.67)	0.847 (0.441-1.628)	0.827 (0.430-1.591)	0.828 (0.429-1.596)	0.748 (0.379-1.474)
Medium or large loss	6 (8.96)	0.455 (0.192-1.080)	0.482 (0.203-1.146)	0.370 (0.154-0.888)	0.373 (0.154-0.906)
Mild gain	22 (26.51)	1.347 (0.793-2.290)	1.419 (0.833-2.418)	1.475 (0.864-2.520)	1.545 (0.896-2.665)
Medium or large gain	28 (25.00)	1.271 (0.776-2.082)	1.500 (0.897-2.508)	1.583 (0.947-2.646)	1.699 (0.993-2.906)

Stable: Weight loss or gain <2 kg. Mild loss: Weight loss 2 to 4 kg. Medium or large loss: Weight loss ≥4kg. Mild gain: Weight gain 2 to 4kg. Medium or large gain: Weight gain ≥4kg.

^a^Absolute body weight change was calculated by the following equations: body weight (2020) - body weight (2012).

Data are RR and 95% confidence intervals (CIs).

Model 1 has been body weight adjusted.

Model 2: Factors from Model 1 as well as baseline gender and age were adjusted.

Model 3: Factors from Model 2 as well as FPG, TG, HDL-c, and SBP at baseline were adjusted.

Model 4: Factors from Model 3 as well as baseline values of TC, LDL-c, BUN, Cr, SUA, ALT, and AST,were adjusted.

## 4 Discussion

In this eight-year retrospective cohort study, we found a strong relationship between weight gain and worsening of metabolism. The incidence of metabolic abnormalities was dramatically increased by long-term, excessive absolute weight gain. This relationship was significant independent of baseline age, sex, and other clinical variables, but it was found only in subjects with non-overweight/obesity. In contrast, long-term absolute weight loss only reduced the risk of metabolic abnormalities in overweight/obese individuals, independent of traditional risk factors. Additionally, the analysis of relative body weight variation produced comparable findings.

Previous studies found an inconsistent relationship between weight change and the risk of metabolic abnormalities in both obese/overweight and non-obese/overweight individuals (11, 23, 29, 30). In a cohort study, the probability of having MetS increased fairly linearly with incremental (1 Kg) weight increases in subjects with normal weight (OR1.18[95%CI1.14-1.21]) (9). A randomized controlled trial (RCT) study with obese people showed that incremental (1 Kg) weight loss, reduced the risk of MetS by 8% (16). However, Lloyd-Jones et al. performed a longitudinal cohort study with young adults and found that maintaining a stable or decreasing BMI over time was associated with a significantly lower incidence of metabolic syndrome. This occurred regardless of whether the subjects had normal weight or overweight at baseline, although an elevated BMI was associated with a higher incidence of metabolic syndrome (13). Conversely, our findings show that the risk of metabolic abnormalities increased with weight gain only in subjects without overweight/obesity and decreased with weight loss only in subjects with overweight/obesity. These differences between the studies are most likely due to the distinctive ethnicity and age of each study population. In our study, long-term absolute weight loss only reduced the risk of metabolic abnormalities in non-overweight/obese individuals, suggesting that non-obese/overweight individuals should keep their weight within the normal range and should not lose excessive amounts of weight, resulting in weight fluctuations. A cross-sectional study indicated that weight fluctuations were more likely to have abnormal metabolic syndrome components among subjects with BMI < 25 kg/m^2^ (31). Moreover, we indicated that a 1 Kg increase in body weight predicted 9.4% and 10.6% of metabolic abnormalities in non-obese/overweight and in overweight/obese subjects, respectively. However, this linear association was not observed when we divided weight change in overweight/obese subjects into five groups. This suggests that the effect of weight change on metabolic health risk is likely to be nonlinear rather than a linear relationship. According to a population-based longitudinal cohort study, those with a greater baseline weight had a lower correlation between weight gain and the number of MetS components (11). An observational study in China also showed an inverted U-shaped association for participants with overweight/obesity: the odds of MetS increased with increasing weight gain, up to approximately 12 Kg, and then decreased (23). These findings are partially consistent with the nonlinear relationships found in this study.

Prior studies have found that the mechanism by which weight gain leads to an increased risk of metabolic abnormalities may be the release of adipokines from adipose tissue, mediating insulin resistance (32–35). However, insulin resistance may not be enhanced with further weight gain in people with obesity because it progressively decreases with longer periods of obesity (36). This may explain the results observed in our study showing that weight gain has a significant impact on metabolic abnormalities in non-overweight or obese people but is insignificant in overweight or obese individuals. In contrast, weight reduction improves insulin resistance in overweight/obese individuals, thereby reducing their risk of metabolic abnormalities (37). Thus, non-overweight/obese individuals should maintain their weight to prevent metabolic abnormalities, whereas people with obesity can mitigate metabolic abnormalities by losing weight. The Chinese guidelines for primary care of obesity (2019) suggest that weight loss of 5–15% or more in patients with obesity significantly improves glycemic control and cardiovascular complications in patients with hypertension, dyslipidemia, and Type 2 diabetes (38). To promote metabolic health, the American College of Cardiology and American Heart Association advise overweight and obese individuals to lose between 5% and 10% of their initial body weight within six months (39).

Our study has several strengths. First, our study was able to simultaneously analyze absolute weight change, relative weight change, and risk of metabolic abnormalities in Chinese adults. Second, the relationship between weight change and risk of metabolic abnormalities was explored in different BMI strata. Third, our study had a long-term follow-up of 8 years. Moreover, the survey replaced self-report and recall weights with measured values and there was no recall bias. However, there were some limitations to this study. First, owing to the lack of baseline data, we were unable to appropriately correct for smoking, drinking, and other characteristics that are relevant to MetS in the risk analysis, despite adjusting for several potential confounding factors, such as age, sex, and other serum biochemical parameter. Second, only those who had information with two-time cutoff points were included. Third, because of the observational design, no causal relationship between weight change and risk of metabolic abnormalities could be determined. Fourth, some possible confounding factors were not collected due to the limitations of retrospective studies, which may have influenced the results to some extent. Therefore, there is an urgent need for further investigations with prospective cohort design. Fifth, the sample size of the study was small.

In conclusion, the risk of metabolic abnormalities associated with weight change was nonlinear and reversible. Therefore, maintaining a BMI within the normal range may help prevent and reduce the risk of metabolic abnormalities.

## Data availability statement

The raw data supporting the conclusions of this article will be made available by the authors, without undue reservation.

## Ethics statement

The studies involving human participants were reviewed and approved by The First Affiliated Hospital’s Clinical Medicine College Ethics Committee at Southwest Medical University. Written informed consent for participation was not required for this study in accordance with the national legislation and the institutional requirements.

## Author contributions

CY and HJ contributed to conception and design of the study. YL and WX organized the database. SY and YD performed the statistical analysis. YD wrote the first draft of the manuscript. All authors contributed to the article and approved the submitted version.
