# Supplementary material for: Association between weight change and risk of metabolic abnormalities in non-overweight/obese and overweight/obese population: A retrospective cohort study among Chinese adults
Source: Front Endocrinol (Lausanne). 2022 Dec 20;13:1029941. doi: 10.3389/fendo.2022.1029941 (PMC9808089; doi:10.3389/fendo.2022.1029941)
Supplement: Supplementary file 1 [file Table_1.docx]

Supplementary Material

# Supplementary Tables

**Table S1. Associations between relative body weight change and risk of metabolic abnormalities**

| Variables | No. of cases(%) | Model 1 | Model 2 | Model 3 | Model 4 |
| --- | --- | --- | --- | --- | --- |
| Relative body weight change^b^ | 203(10.71) | 1.016(1.000-1.033) | 1.033(1.016-1.051) | 1.047(1.029-1.065) | 1.057(1.038-1.077) |
| Stable | 59(9.59) | 1 | 1 | 1 | 1 |
| Mild loss | 19(10.56) | 1.100(0.656-1.845) | 0.968(0.577-1.627) | 1.093(0.649-1.841) | 1.025(0.605-1.737) |
| Medium or large loss | 11(7.14) | 0.745(0.391-1.417) | 0.722(0.379-1.376) | 0.611(0.321-1.165) | 0.559(0.292-1.072) |
| Mild gain | 47(14.24) | 1.485(1.012-2.178) | 1.647(1.121-2.420) | 1.677(1.137-2.473) | 1.824(1.229-2.706) |
| Medium or large gain | 67(10.88) | 1.134(0.799-1.609) | 1.498(1.044-2.148) | 1.848(1.280-2.670) | 2.044(1.404-2.978) |

Note: Stable: loss or gain of 3%. Mild loss: loss of 3 to 6%. Medium or large loss: loss of ≥ 6%. Mild gain: gain of 3 to 6%. Medium or large gain: gain ≥ 6%.

b Relative body weight change was calculated by the following equations: (body weight at 2020 - body weight at 2012) / body weight at 2012

Data are RR and 95% confidence intervals (CIs).

Model 1 has been body weight adjusted.

Model 2: Factors from Model 1 as well as baseline gender and age were adjusted.

Model 3: Factors from Model 2 as well as FPG, TG, HDL-c, and SBP at baseline were adjusted.

Model 4: Factors from Model 3 as well as baseline values of TC, LDL-c, BUN, Cr, SUA, ALT, AST, and BMI were adjusted.

**Table S2. Associations between relative body weight change and risk of metabolic abnormalities in subjects with non-overweight/obesity.**

| Variables | No. of cases(%) | Model 1 | Model 2 | Model 3 | Model 4 |
| --- | --- | --- | --- | --- | --- |
| Relative body weight change^b^ | 99(7.18) | 1.027(1.006-1.050) | 1.043(1.020-1.066) | 1.049(1.026-1.072) | 1.055(1.030-1.080) |
| Stable | 20(4.73) | 1 | 1 | 1 | 1 |
| Mild loss | 8(7.21) | 1.524(0.671-3.461) | 1.356(0.595-3.091) | 1.781(0.765-4.146) | 1.579(0.662-3.762) |
| Medium or large loss | 5(5.56) | 1.175(0.441-3.131) | 1.001(0.374-2.680) | 1.198(0.436-3.293) | 0.961(0.349-2.643) |
| Mild gain | 24(9.96) | 2.106(1.164-3.813) | 2.379(1.311-4.317) | 2.315(1.264-4.241) | 2.181(1.178-4.038) |
| Medium or large gain | 42(8.19) | 1.732(1.017-2.949) | 2.177(1.267-3.740) | 2.628(1.500-4.605) | 2.491(1.422-4.363) |

Note: Stable: loss or gain of 3%. Mild loss: loss of 3 to 6%. Medium or large loss: loss of ≥ 6%. Mild gain: gain of 3 to 6%. Medium or large gain: gain ≥ 6%.

b Relative body weight change was calculated by the following equations: (body weight at 2020 - body weight at 2012) / body weight at 2012

Data are RR and 95% confidence intervals (CIs).

Model 1 has been body weight adjusted.

Model 2: Factors from Model 1 as well as baseline gender and age were adjusted.

Model 3: Factors from Model 2 as well as FPG, TG, HDL-c, and SBP at baseline were adjusted.

Model 4: Factors from Model 3 as well as baseline values of TC, LDL-c, BUN, Cr, SUA, ALT, and AST were adjusted.

**Table S3. Associations between relative body weight change and risk of metabolic abnormalities in subjects with overweight/obesity.**

| Variables | No. of cases(%) | Model 1 | Model 2 | Model 3 | Model 4 |
| --- | --- | --- | --- | --- | --- |
| Relative body weight change^b^ | 104(20.12) | 1.038(1.010-1.067) | 1.054(1.021-1.088) | 1.068(1.034-1.103) | 1.076(1.040-1.113) |
| Stable | 39(20.31) | 1 | 1 | 1 | 1 |
| Mild loss | 11(15.94) | 0.785(0.402-1.532) | 0.759(0.388-1.484) | 0.768(0.392-1.508) | 0.722(0.361-1.446) |
| Medium or large loss | 6(9.38) | 0.462(0.195-1.090) | 0.500(0.211-1.189) | 0.379(0.158-0.910) | 0.379(0.156-0.919) |
| Mild gain | 23(25.84) | 1.272(0.760-2.130) | 1.329(0.793-2.227) | 1.448(0.860-2.438) | 1.514(0.889-2.578) |
| Medium or large gain | 25(24.27) | 1.195(0.123-1.974) | 1.464(0.858-2.497) | 1.610(0.940-2.757) | 1.724(0.988-3.010) |

Note: Stable: loss or gain of 3%. Mild loss: loss of 3 to 6%. Medium or large loss: loss of ≥ 6%. Mild gain: gain of 3 to 6%. Medium or large gain: gain ≥ 6%.

b Relative body weight change was calculated by the following equations: (body weight at 2020 - body weight at 2012) / body weight at 2012

Data are RR and 95% confidence intervals (CIs).

Model 1 has been body weight adjusted.

Model 2: Factors from Model 1 as well as baseline gender and age were adjusted.

Model 3: Factors from Model 2 as well as FPG, TG, HDL-c, and SBP at baseline were adjusted.

Model 4: Factors from Model 3 as well as baseline values of TC, LDL-c, BUN, Cr, SUA, ALT, and AST were adjusted.
